# Lab on a Chip Device for Diagnostic Evaluation and Management in Chronic Renal Disease: A Change Promoting Approach in the Patients’ Follow Up

**DOI:** 10.3390/bios13030373

**Published:** 2023-03-12

**Authors:** Margherita Borriello, Giuseppe Tarabella, Pasquale D’Angelo, Aris Liboà, Mario Barra, Davide Vurro, Patrizia Lombari, Annapaola Coppola, Elvira Mazzella, Alessandra F. Perna, Diego Ingrosso

**Affiliations:** 1 Department of Precision Medicine, University of Campania “Luigi Vanvitelli”, via L. De Crecchio, 7, 80138 Naples, Italy; 2 IMEM-CNR, Parco Area delle Scienze 37/A, 43124 Parma, Italy; giuseppe.tarabella@imem.cnr.it (G.T.);; 3 CNR-SPIN, c/o Dipartimento di Fisica “Ettore Pancini”, P.le Tecchio, 80, 80125 Naples, Italy; 4 Department of Translational Medical Science, University of Campania “Luigi Vanvitelli”, via Via Pansini, Bldg 17, 80131 Naples, Italy

**Keywords:** CKD, Lab on a Chip, POCT, microfluidics, biosensors, cardiovascular disease, uremic toxins detection, quality control assurance

## Abstract

Lab-on-a-chip (LOC) systems are miniaturized devices aimed to perform one or several analyses, normally carried out in a laboratory setting, on a single chip. LOC systems have a wide application range, including diagnosis and clinical biochemistry. In a clinical setting, LOC systems can be associated with the Point-of-Care Testing (POCT) definition. POCT circumvents several steps in central laboratory testing, including specimen transportation and processing, resulting in a faster turnaround time. Provider access to rapid test results allows for prompt medical decision making, which can lead to improved patient outcomes, operational efficiencies, patient satisfaction, and even cost savings. These features are particularly attractive for healthcare settings dealing with complicated patients, such as those affected by chronic kidney disease (CKD). CKD is a pathological condition characterized by progressive and irreversible structural or functional kidney impairment lasting for more than three months. The disease displays an unavoidable tendency to progress to End Stage Renal Disease (ESRD), thus requiring renal replacement therapy, usually dialysis, and transplant. Cardiovascular disease (CVD) is the major cause of death in CKD, with a cardiovascular risk ten times higher in these patients than the rate observed in healthy subjects. The gradual decline of the kidney leads to the accumulation of uremic solutes, with negative effect on organs, especially on the cardiovascular system. The possibility to monitor CKD patients by using non-invasive and low-cost approaches could give advantages both to the patient outcome and sanitary costs. Despite their numerous advantages, POCT application in CKD management is not very common, even if a number of devices aimed at monitoring the CKD have been demonstrated worldwide at the lab scale by basic studies (low Technology Readiness Level, TRL). The reasons are related to both technological and clinical aspects. In this review, the main technologies for the design of LOCs are reported, as well as the available POCT devices for CKD monitoring, with a special focus on the most recent reliable applications in this field. Moreover, the current challenges in design and applications of LOCs in the clinical setting are briefly discussed.

## 1. Introduction

In recent years, a growing scientific interest has emerged in the applications of Lab on a Chip (LOC) devices in biomedicine. LOC systems are miniaturized devices aimed to perform one or several analyses, normally carried out in a laboratory, in a single chip. LOC systems have a wide application range [1,2], including diagnosis (COVID-19) [3], clinical biochemistry (glucose, INR, blood gases measurements), nucleic acid detection [4], cell biology research (organ on a chip) [5], biomedical tissue engineering, environmental sampling, food safety [6,7], natural products discovery, and overproduction [8].

From the clinical point of view, LOCs belong to Point of Care Testing (POCT), the driving concept of which is to provide lighter, compact, and agile instrumentation, as well as devices suitable for performing tests in a rapid and unobtrusive way. The recent coronavirus pandemic has underlined the need to move from traditional lab-centralized diagnostics to LOC settings. In addition to the pandemic, significant changes have affected the healthcare system. These changes are driven by an increasingly patient-centered approach, a greater emphasis on primary care, ever faster and more efficient patient triage, a re-thinking of the hospital as a single care center, a reduction in hospitalization time and the reorganization of hospitals. In this view, LOC systems represent a model for a clinical lab reorganization, which offers many advantages in POCT applications. The use of LOCs in the clinical setting circumvents several of the steps required in central-core lab testing, including specimen transportation and processing, resulting in faster turnaround times (TAT) [9,10,11].

The development of POCTs requires collaboration between different expertise, such as clinicians, laboratorists, technicians, and experts in computation fluid dynamics, microfluidics, bioelectronics, and material science. Irrespective of the multi-disciplinary approach, POCTs have numerous advantages, such as a low volume sample requirement, a reduction in chemicals use and, as a consequence, a reduction in potentially dangerous wastes, ease of use and compactness, no environmental limitations, and relatively low-cost. All of these features match well with the World Health Organization guidelines to develop a POCT system (Table 1) [12].

With the advent of the digital age, the ASSURED guidelines have been updated: from ASSURED criteria to REASSURED. The update emphasizes the importance of the Real-time connectivity (“R”), and Ease of specimen collection (“E”) [13].

The features and the advantages related to POCT are particularly favorable for critical patients suffering from serious medical conditions such as chronic kidney disease (CKD). CKD is a syndrome delineated as alterations in kidney function and/or structure characterized by the loss of nephrons and renal fibrosis and lasting more than three months. CKD is an important health care problem, certainly compared to diabetes, for its scope, impact, and consequences on well-being. Between 8 and 16% of the world population is affected by CKD, representing the sixth largest cause of death worldwide [14]. Cardiovascular disease (CVD) is the major cause of death in CKD, with a cardiovascular risk ten times higher in these patients than the rate observed in healthy subjects. The gradual decline of the kidney leads to the accumulation of uremic solutes, with a negative effect on organs, and particularly on the cardiovascular system [14]. Monitoring CKD patients by non-invasive and low-cost approaches could give advantages both to the patient outcome and to the sanitary costs. Indeed, POCT-assisted patient management possesses the potential to improve clinical outcomes and patient quality of life and to increase patient participation with their disease management [15]. In this regard, various studies have reported that patients under peritoneal dialysis and home dialysis have declared an improved satisfaction and comfort, with the suggestion of improved outcomes [16,17,18].

Despite the numerous advantages, many challenges still affect the spreading of POCT in the overall clinical setting, not only in CKD management. One of the main challenges is managing the data generated by POCT. When tests are performed at the point-of-care, it is important that the results are incorporated into the patient’s medical record. An important issue is the absence of standards and guideline specific for POCTs, as well as the difficulty to translate the quality control assurance to POCTs. These issues represent the most critical aspects affecting the spread of LOCs in clinical practices. [19,20]. Scientific communities from different countries have published standards for accreditation, which include guidance for users of the technology, key quality requirements for POCT performance, and measures to warrant the safety and quality of the test results. The guarantee indicators that could be monitored include accuracy in patient identification, turnaround time, skills assessment of POCT professionals, sample acceptability for testing, POCT device defects, reporting of critical results, accidents with percutaneous devices, and the ratio of all patient test results that are correctly transcribed or inserted into a patient’s file [11,21]. Moreover, well-trained users need to be alert in analyzing the results and communicating concerns regarding the device and patient factors, while ensuring sufficient patient training on the use of their devices. Hence, the work required in the implementation of a new POCT pathway could be enormous and involves a transformation of diagnostic services and care provision [22,23].

Thousands of POCT devices have been proposed, but only few are able to analyze untreated samples and involve processes that make them suitable for home use. A small percentage of these devices have been commercialized and only a few of these have been successfully evaluated and integrated into clinical practice [24]. This review describes the main technologies for POCT/LOC design, as well as the available POCT devices used for CKD monitoring, with a special focus on the most recently published papers in this field. Moreover, the current technological and clinical challenges to the spread of LOCs in clinical the setting, with a special focus on CKD management, are briefly discussed.

Specifically, this review is organized as follows: Section 2 covers the LOCs’ design, the most important technologies used for LOC manufacturing, and the main methods for the molecular target detection. Section 3 and Section 4 describe the main clinical features of CKD, with a focus on the biochemical features, and the utility of LOCs’ application in CKD management. Section 5 and Section 6 include the current challenges in the spread of LOCs in the clinical setting and discuss the possible perspectives to implement future work in this field [25].

## 2. LOCs Design, Available Technologies, and Detection Methods

In order to fully exploit the LOCs’ related advantages, many aspects need to be optimized in their design, fabrication, and assembly. Many of these issues are specifically related to the employed materials and the detection mechanisms of the LOC system, which still require further research and optimization. In this section, an overview of the main techniques and technologies applied for the fabrication of LOCs is provided.

### 2.1. LOCs-Related Technologies

**Figure 1:**
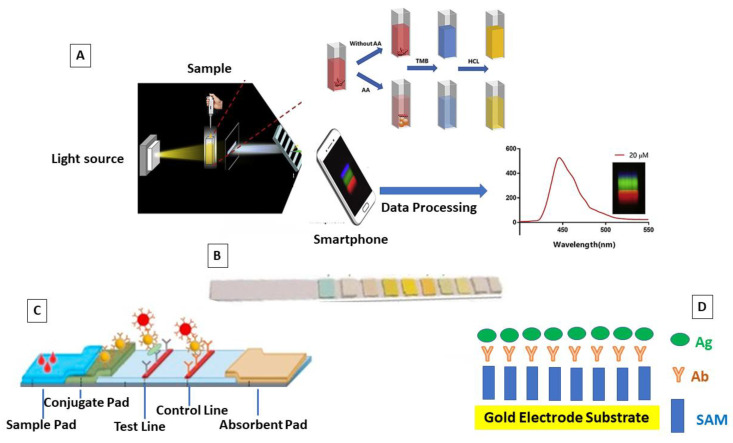
Schematic representation of the LOCs technologies. (**A**) Cuvettes-containing Reagents. The reagents are pre-stored in the cuvettes, so, once sample is added, a color change can be observed. (**B**) Dipsticks are directly soaked by specific reagent. (**C**) Lateral Flow Assay (LFA) system is characterized by the presence of an antibody (represented by “Y” in the figure) that specifically bind the target molecule. (**D**) Schematic representation of biosensor recognition interface. The biorecognition element can be an aptamer, antibody, enzyme, receptor. Figure adapted from [27] for panel A, Ref. [28] for panel B, Ref. [29] for panel C, with kind permission from Elsevier.

LOC systems are handheld, and are thus completely portable devices. These systems can be classified into four classes based on different technologies: (A) cuvettes-containing reagents; (B) dipsticks; (C) lateral flow assays; (D) electronic/electrochemical/optical biosensors (Figure 1) [26].

The first three technologies exploit a direct colorimetric/optical transduction, while the latter also makes use of an electronic transduction, promoting its suitability for direct integration into electronic platforms able to accomplish data transmission, manipulation, and analysis within an Internet of Things (IoT) context.

Cuvettes-containing reagents are colorimetric tests in which the reagents are stored in the test tube. Once the sample is added, a change in the color is produced. The entity of the color change is directly proportional to the analyte concentration [30].

Dipsticks are directly impregnated with reagents. The sample addition generates a color change. The reaction is read by eyes or helped by the use of a reflectance meter. Each square allows the detection of different molecules based on the related reagents in the dipstick area [30].

Lateral flow assay (LFA) can be considered the most known LOCs technology. LFA is based on the antigen-antibody binding on a strip; therefore, LFA can be defined as an immunochromatographic method. Briefly, the antigen moves through the strip (for capillarity) and, when it “finds” the related antibody, a binding between the two molecules occurs. The color produced into the strip is due to the conjugation of the antibody with a chromogen molecule: only following the antigen-antibody binding, the appearance of the color occurs.

As in LFA devices, biosensors also take advantage of the antigen-antibody binding. Nevertheless, LFA devices and biosensors differ from each other in terms of their detection scheme, with the latter exploiting surface plasmon resonance or electrochemical methods. Biosensor can be defined as a device incorporating a sensing layer of biological entities (e.g., enzymes, antibodies, DNA fragments, nanobodies) that specifically bind a certain target molecule. The interaction with the target produces a measurable electrical or optical signal by means of the transducer element [31]. Recently, aptamers-based biosensors have been shown in the literature, offering some advantages over the antigen-antibody in terms of selectivity [32]; the non-covalent binding of aptamers also allows multiple cycling measurements to be performed in the same sample, by changing, for example, the pH of the solution by dilution. [33].

The first definition of biosensor was given by Leland Charles Clark Jr., in 1962, who had the idea to integrate two components, i.e., a bioreceptor and a transducer, into a single device. Clark Jr also demonstrated the use of an amperometric enzyme electrode for glucose sensing [34].

### 2.2. Techniques for LOC Devices Fabrication

**Figure 2:**
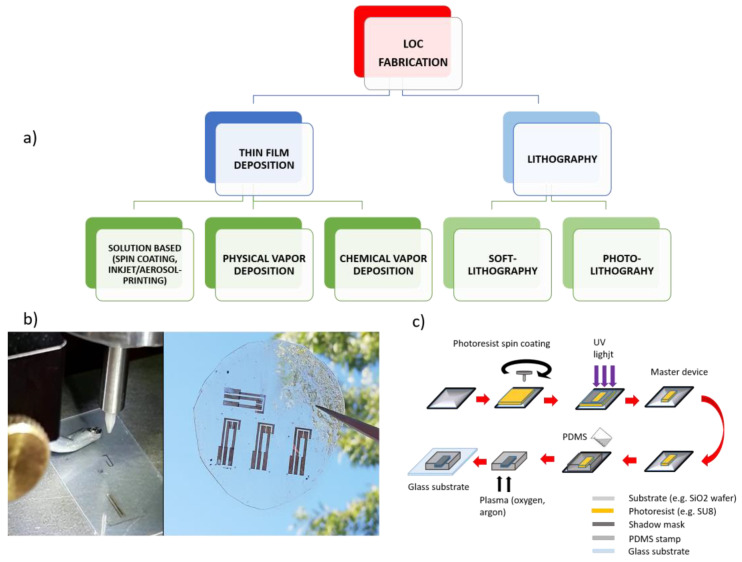
(**a**) Main fabrication techniques here discussed for LOCs Fabrication; (**b**) 3D printing techniques (detail of an AJP nozzle and printed silver contacts on a flexible parylene C substrate); (**c**) combination of photolithography and soft lithography for microfluidics manufacturing.

As the LOC chips are highly compact devices characterized by very small components, various microfabrication techniques have been applied for their development. On the basis of their operation principles, these techniques can be mainly classified as additive and subtractive techniques (Figure 2a) [35].

Additive techniques are defined as “ground to top” approaches as, in this case, the chip structures are manufactured through a layer-by-layer approach. Thin film deposition techniques belong to this category, as the functional materials used to fabricate the transducer element of several types of biosensors are deposited in the form of thin coatings (i.e., thickness from the nanoscale to the microscale) on proper substrates. At present, several types of deposition techniques are available, with advantages or disadvantages, according to the specific application and related requirements [36].

Examples of thin film deposition techniques are: chemical vapor deposition (CVD), physical vapor deposition (PVD), thermal oxidation, spin-coating, and printing process. In CVD, the precursor of the material to be deposited is taken in gaseous form in order to interact with the substrate surface and to accordingly produce the final coating. While the reaction between the gases and catalysts take place, the final material of interest is formed, while the useless by-products are removed by the gas flow [37].

In PVD, the desired material is initially taken in its solid form, then it is vaporized by providing an adequate amount of energy. The specific source of energy supply and the related interaction with the target substrate causing the material vaporization is peculiar of any technique [38]. The main deposition parameters are related to the amount of the vaporized material impinging on the substrate per unit area and time (i.e., deposition rate) and to the substrate temperature during the film formation. Both the CVD and PVD techniques are applied to a wide number of materials and are mainly performed in vacuum chambers in order to guarantee the high compositional purity of the final layer [39].

In particular, among the various PVD processes, those providing the deposition of thin metal films are of fundamental importance for the fabrication of conducting electrodes or other functional layers to be integrated in the main components of the LOC devices. In this context, the two main approaches are related to the thermal evaporation and the sputtering technique [36]. In the thermal evaporation process, metals, mostly in the form of wires and pellets, are located in specific evaporation sources, called “boats” and are usually made from various refractory materials (e.g., tungsten, tantalum, molybdenum or ceramic-type) being able to sustain very high temperatures. Usually, these thermal sources are connected to external massive copper feedthroughs (cooled by water) and the evaporation process is initiated through the flow of a high current (up to a few hundreds of Amps) in order to heat up the material of interest until it reaches its sublimation temperature. A more sophisticated version of the thermal evaporation is given by the so-called electron-beam method. Electron beam evaporation relies on the use of cathodes, which are heated in such a way to emit a high flux of electrons accelerated by the application of large voltages (up 15 keV). Through proper magnetic systems, the electron beam is deflected by the Lorentz force and is finally focused on a crucible containing the material to be deposited; this is vaporized and ends up condensing on the substrate where the final coating is formed. As electron beam evaporation allows higher temperatures than the conventional thermal approach to be achieved, it assures very fast and highly controlled deposition rates for a wider range of materials.

Another technique that is commonly used for the deposition of metals or alternative materials with more complex stoichiometry is sputtering [36]. The main quality of this process is given by the possibility to deposit materials with very high melting points, which could be barely deposited by evaporation. Moreover, sputter-deposited films maintain a chemical composition that is very close to that of the initial source material, and their adhesion level on the substrate is significantly improved in comparison to the evaporated layers. Sputter deposition takes place in an evacuated chamber where a low pressure (few mTorr) of a noble (e.g., argon) is introduced. When a voltage is applied between a metal target (the source of the material to be deposited) and the final substrate, the gas molecules are ionized and a plasma is formed inside the chamber. In this way, the positively charged ions are accelerated and are able to remove the target atoms through collision and momentum transfer. Then, the emitted species condense on the substrate surface and the final film is produced. Sputtering systems with different characteristics can be achieved by the proper choice of the power supply systems, which can work in DC, pulsed DC, or RF regimes. In a magnetron sputtering system, very strong magnets are employed to confine the electrons in the plasma at or close to the target surface. This feature has a significant impact as it allows increased deposition rates and prevents the damages that can be produced by the impact of these electrons with the surface of the substrate or the depositing film.

3D printing is an additive-based manufacturing technique with high potential in rapid device prototyping. Among the various 3D printing methods, the most suitable for the production of LOC devices are the Direct-Writing, non-contact techniques, inkjet and aerosol jet printing (Figure 2b). Such techniques require the availability of proper material solutions and/or stable suspensions [34,40,41,42] to be used as inks for in-line device prototyping. They allow the possibility of using a software for the design of the printing patterns [41], resulting in a number of advantages, such as limited material consumption and printing on non-flat and wearable substrates. Screen printing, roll-to-roll, nanoimprinting, gravure printing, and transfer printing are popular techniques that exploit a contact mode to define the device parts of bioelectronics devices [42,43,44].

Spin coating represents probably the easier method to deposit a thin film on a planar substrate, provided the solubility of the starting material is in a proper solvent [45]. In this case, a solution of the material is poured on the substrate surface, which is then rotated at considerable speeds, typically ranging between hundreds and several thousands of rpm (revolutions per minute). Under the action of the centrifugal force, the coating material is spread on the entire substrate surface. Beyond the characteristic features of the starting solution (i.e., solvent type, concentration, etc.), the typical deposition parameters are associated with the velocity, related accelerations, and the final duration of the entire spinning process. After the evaporation of any remaining solvent, which can be also favored by post-deposition annealing procedures, uniform coatings with thicknesses going ranging between nanometers and a few microns can be achieved. In many cases, similarly to the other solution-based techniques, the spinning processes can be carried out in controlled atmospheres (e.g., nitrogen regulated glove boxes) in order to hamper the contamination effects during the phase of the film formation [38]. For device prototyping, spin coating is used in combination with lithographic techniques to define the required device interfaces.

Subtractive techniques for LOCs production mainly refer to etching processes, which can be applied in dry or wet modes. Wet etching always uses chemical solutions to selectively etch the target material. Conversely, dry etching can be also performed in the presence of physical agents (e.g., as ions, electrons, etc.), allowing the removal of the target material [46]. Many of the presently applied etching processes belong to the category of “lithographic techniques”, with a resolution down to the atomic scale [47]. The original concept of lithography was developed by Alois Senefelder in 1796. Senefelder designed a printing method to imprint some features on planar surfaces upon pre-printing them using hydrophilic media affined to the post-deposited ink. Still, today, most books are printed using offset lithography, the most common form of printing production. In the 20th century, the semiconductor industry developed new sophisticated lithography-inspired techniques to fabricate highly miniaturized devices and related integrated circuits (ICs), opening the way to the rise of modern electronics [48]. In particular, photolithography (Figure 2c) uses a beam of light to transfer a pattern written on an optical mask to the substrate surface of interest. This technique consists of a large number of steps and relies on the fundamental role of photo-sensitive polymers, called photoresists, which are previously deposited on the substrate through the spin-coating process. When a proper light beam passing through the mask is focused on the substrate, the physico-chemical structure of the photoresist regions hit by the light is modified. According to the specific type of photoresist, the following etching processes allow the selective removal of the substrate regions, which are not included in the desired final pattern. As a result of the diffraction limit, the minimum feature size achievable by this technique is limited to half of the incident wavelength, thus requiring the adoption of ultra-violet radiation to further improve the final resolution. An even smaller size, down to the range of few nanometers, can be achieved by using ion or electron beams as alternative to photons [49], but also electrical methods [50].

Finally, it should be outlined that a new lithographic technique, defined as soft-lithography and commonly involving some photolithographic steps, has been developed in the last few decades, as has been extensively employed in biomedical research. In this case, the desired pattern is transferred in elastomeric stamps through the use of specialized photoresists that can be deposited in the form of films, with an accurate control of the related thickness up to hundreds of microns. Such a technique is often carried out in combination with photolithography to develop microfluidics devices. Soft-lithography is a powerful technique with crucial importance for the development of microfluidic systems, an essential part of LOC devices in several applications [51,52,53], and for the fabrication of biomaterial micropatterns to study the cell–biomaterial interaction [50,54].

All of the above techniques are used to fabricate a large set of devices on different substrates, also allowing the fabrication of 3D artefacts and the integration of biosensor devices in existing or novel platforms by means of 3D approaches [53,55], as well as in wearable solutions [56], by exploiting specific functionalization strategies. The materials used to fabricate LOC devices range between metals for interconnections and electrodes in electronic biosensors and solution processable insulating and conducting organics to fabricate, for instance, microfluidics, reaction chambers, and dielectric interfaces with the former, as well as electronic transducers with the latter. Substrate materials may range between conventional glasses and oxides, such as silicon dioxide and silicon nitride, and plastic ones, which are flexible and sometimes stretchable, hence allowing the production of comfortable and bendable devices. Plastic substrates, such as kapton and polyethylene terephthalate (PET), offer advantages in terms of their biocompatibility and are comparable to the lithographic steps [57], while stable coatings, such as parylene C, are suitable for printing by DW methods, determining a good quality of the printed lines. 3D printing fabrication methods known as “solid freeform fabrication” have also promoted the use of bioecofriendly, even natural, such as polylactic acid (PLA), while biopolymers such as woven silk, thin silk fibroin films and woven-non-woven fabrics (fabricated, for instance, by electrospinning) are currently emerging as substrates for wearable sensors [58].

### 2.3. Detection Methods for LOC Devices

LOC systems operate in the presence of small sample volumes, and the employed detection methods play a fundamental role in assessing a proper response upon processing such samples. Indeed, the availability of very accurate and sensitive detection methods is mandatory to preserve the advantages related to LOC use, i.e., rapid response, minimal sample preparation, and low fabrication costs. To this end, different detection approaches have been developed relying, mainly, on electrochemical, mechanical, and optical methods, as briefly summarized in the following section (Table 2).

- Electrochemical: Electrochemical detection involves the interaction between chemical species and electrodes or probes. This interaction results in changes in the electrical signals. Different parameters can be measured, such as changes in the conductance/resistance due to the redox activity involving biological species and/or capacitance due to the electrical double layer formation at the active surface of the electrodes. The main advantages related to electrochemical detection are the possibility to improve a real time detection through low-cost electrodes. For these reasons, they are widely used in POCTs. On the other hand, the main drawbacks regard the need and the difficulty to control the ionic species concentration before detection, the complex and potentially ambiguous character of data analysis and interpretation, and, finally, the short shelf life of the electrochemical systems [59,60].
- Mechanical: Mechanical systems generally refer to the use of micro-cantilevers. The detection is based on variations in the resonant frequency or surface stress of the mechanical sensor. Cantilever-based devices work in two different ways: (i) static deflection, where binding on one side of a cantilever causes unbalanced surface stress, resulting in a measurable deflection; (ii) dynamic mode, where binding on a cantilever causes variations in its mass, and consequently shifts the resonant frequency. The main advantage of mechanical detection is the label-free detection; the main disadvantages are the long response time (around 30 min) and the complex fabrication [61].
- Optical: Among the detection methods, optical detection is likely the most suitable for LOCs because they offer the optimal compromise between the sensitivity and specificity of the detection. This method measures the variations in the light intensity, refractive index, or fluorescence intensity. Optical detection is characterized by negligible sample preparation, minimal interferences from artifacts in respect to the electrochemical methods, and real-time results. However, the employed opto-instrumentation is usually quite expensive [62]. Significantly, the high accessibility of smartphones and their improved technological features (cameras, connectivity, and computational power) have allowed smartphone integration with a wide range of analytical systems [63]. Accordingly, the use of smartphones’ sensors provides advantages not only in terms of lightweight and more affordable solutions, but also in terms of the abilities to process images by means of dedicated applications and to implement a wireless data sharing for a real time data analysis by remote computing [64]. Detection via smartphone is commonly based on various forms of optical measurements, including bright-field, colorimetric, luminescence, and/or fluorescence [27,65,66].

## 3. Chronic Kidney Disease

CKD is a syndrome delineated as alterations in kidney function and/or structure lasting more than three months, characterized by the loss of nephrons and renal fibrosis. The disease is also aggravated by a high cardiovascular risk, representing the major cause of death. The disease, classified into five stages according to the glomerular filtration rate (GFR) and albuminuria, (Table 3), displays an unavoidable tendency to progress to End Stage Renal Disease (ESRD) (uremia), thus requiring renal replacement therapy, usually dialysis, or kidney transplantation [14].

Renal failure can be revealed and monitored by finding biochemical abnormalities. Historically, the gold standard for monitoring kidney function is creatinine detection. Moreover, the Glomerular Filtration Rate (GFR), elevated plasma creatinine or urea concentration, or the presence of protein in urine represents the main biochemical investigation performed to monitor kidney diseases progression [67]. As mentioned above, CKD patients are subjected to a broad range of complications, and particularly cardiovascular disease (CVD). Several mechanisms are involved in the progression of CKD toward ESRD, which are still not entirely clear. For example, the accumulation of uremic toxins, and especially sulfur compounds such as homocysteine and lanthionine, are among the leading culprits affecting cardiovascular risk [68]. Many clinical manifestations are related to CKD. In terms of the cardiovascular system in CKD, hypertension, congestive cardiac failure, and accelerated atherosclerosis are all frequently encountered. Neurological involvement can include both the central nervous system (uremic encephalopathy) and the peripheral nervous system. The features of the bones in CKD comprise the deregulation of calcium and phosphate metabolism (with increased vascular calcification), osteomalacia, osteosclerosis, and osteopenia. These overall manifestations are known as «chronic kidney disease-bone mineral disease» (CKD-BMD). CKD-MBD is strictly related to an impaired production of calcitriol, the hormone derived from vitamin D [68,69,70]. CKD is also associated with gastrointestinal problems, (hiccoughs, anorexia, gastritis, gastrointestinal bleeding, alteration in intestinal barrier permeability), which are also related to dysbiosis. Dysbiosis, in turn, is related to an increased production of gut-derived toxins and altering the intestinal epithelial barrier [71]. These changes can lead to an acceleration of the process of kidney injury [72]. In addition, anemia is a common complication of CKD [73]. Moreover, alterations in the acid-base, as well as in the electrolytes metabolism occur in CKD.

CKD affects more than 10% of the general population worldwide, amounting to >800 million individuals. Dialysis cost covers at least 2% of the overall healthcare expenditure for only 0.1–0.2% of the general population, resulting in CKD being one of the most costly non-communicable diseases [68,72,74]. The altered environment that characterizes CKD induces the accumulation of modified amino acid residues in proteins, interfering with normal protein structure and activity [75,76].

This situation carries weight in both public health organization and sanitary costs. The possibility to monitor these patients by using non-invasive and low cost approaches could give advantages both to the patient outcome and to the sanitary costs. Moreover, POCT performed by the patient at home offers a wealth of opportunities to develop individualized, empowering clinical pathways [77].

## 4. CKD Management and Available POCT

In light of the previous section, it is clear that many biochemical parameters could be monitored by LOC systems in patients with renal diseases. In Table 4, the main, already commercially available, POCTs that are useful for CKD are reported, with reference to the setting of application (home or not).

The reported POCTs are only few in number compared to those used and useful for other CKD clinical manifestations. Moreover, considering both the scientific and social/economic impacts of the spread of POCTs—especially for critic patients—various LOCs systems are being programmed, with the aim of improving the evaluation of renal disease progression. The quantification of electrolytes, urea, and creatinine is very important for CKD patients. However, the available POCTs for the measurements of these parameters are not currently authorized for home use [21]. The reason for this is related to possible interference due to the complexity of the creatinine specimen matrix, as well as the haemolysis associated with finger-pricking, which makes a potassium measurement almost impossible via the POCT-associated method [84]. Very recently, Yonel Z. and coworkers determined the concordance of three POCT devices, i.e., (Nova StatSensor (creatinine/eGFR); Siemens DCA-Vantage (HbA1C); CityAssays (vitamin-D), with laboratory-based standard assays employed within clinical biochemistry laboratories. The authors found that POCT devices demonstrate a good concordance with laboratory testing, with at least 95% of all samples being within two standard deviations for each of the devices tested [85]. On the other hand, the detection performance of POCTs need to be improved in terms of the variability in precision between the large number of devices available. Indeed, the inter-variability has also impacted the spread of POCTs [85]. Li J. and co-workers developed a nano-integrated biosensor for taking creatinine measurements. In particular, gold nanostructure- and carbon nanotube-based screen-printed carbon electrodes were integrated into a microfluidic system, enhancing the detection and eliminating many interferences [86]. One of the more recently developed POCTs was applied for the quantification of albumin in urine [87]. The excretion of albumin in urine is called albuminuria. Albuminuria is a reliable indicator of many human diseases, including kidney disease, cardiovascular disease, and diabetes [88]. Vutthikraivit et al. (2021) developed and validated an LFA-based biosensor for detecting and measuring albumin in the urine of CKD patients. To this end, monoclonal antibodies against human serum albumin (HSA) were picked from the ibridomas of spleen cells from immunized mice. This LOC system was tested by using urine samples from CKD and type I and II diabetes patients and the results were compared to both the available POCTs and gold standard methods for albumin detection. The LFA-based biosensor developed by Vutthikraivit showed a sensitivity of 86%, a specificity of 94%, and a positive predictive value of 96% [89]. Another valuable POCT device designed for urine albumin detection is an electrochemical biosensor. Specifically, Tseng and colleagues developed a screen printed electrode and a double-layer reagent paper detection zone impregnated with amaranth. In the proposed device, amaranth (an electroactive substance) is adsorbed on the surface of the electrode under the effects of an external potential and subsequently reacts with the albumin content in the urine sample. The reaction process results in the formation of an inert layer on the electrode surface, which leads to a reduction in the response current, from which the albumin concentration can then be inversely derived [89].

Cardiovascular disease (CVD) is the major cause of death in CKD patients and the traditional risk factors insufficiently explain the high risk for CVD in CKD. Recently, much attention has been paid to the non-traditional risk factors, such as uremic toxins, inflammation, endothelial dysfunction, oxidative stress, and dysbiosis [68,90]. Uremic toxins, such as advanced glycation end products (AGEs), p-cresol, and lanthionine, strongly contribute to endothelial damage and maintain a sustained inflammatory state [90,91,92]. For this reason, the development of new LOC devices for the analysis of uremic toxins and inflammatory markers will improve CKD management and CKD patient quality of life. In this view, Moradi and colleagues recently developed a new simple method for p-cresol estimation. The published method is based on the fluorescence spectroscopy technique and was tested by using plasma samples derived from CKD patients. The developed method showed repeatability, selectivity, and accuracy; moreover, the method has great potential for developing new POCT devices [93]. To the best of our knowledge, no LOC devices have been designed for uremic toxins quantification.

## 5. Current Limitations to LOCs Spreading in Clinical Setting

Despite these numerous advantages, several challenges still affect the development of LOCs. Firstly, the LOC-associated research focuses on different aspects in respect to the requirements for the design and production of LOCs. The research about the industrialization of LOC technologies includes the adaptation of fabrication processes, the design of specific surface treatments, flow control system, the optimization of the suitable techniques, balancing material costs, and the yield. Moreover, quality control, connectivity, and the draw-up univocal guidelines for LOC use and management have priority for industrialization [11,94]. Indeed, one of the main challenges concerns the managing of the data generated by POCTs. When tests are performed at the point-of-care, it is important that the results are incorporated into the patient’s medical record [20]. Scientific societies of different countries have published standards for accreditation, which include guidance for users of the technology, key quality requirements for POCTs performance, and measures to guarantee the safety and quality of the test results. The guarantee indicators that could be monitored include the accuracy in patient identification, turnaround time, skills assessment of POCT professionals, sample acceptability for testing, POCT device defects, reporting of critical results, accidents with percutaneous devices, and the ratio of all the patient test results that are correctly transcribed or inserted into a patient’s file [20,21]. Appropriately trained staff need to be carefully supervised in reviewing the results and communicating their concerns regarding the device and patient factors, while ensuring sufficient patient training on the use of their devices. At present, the scientific community is devoting a lot of energy to overcome these issues. In this regard, an example is given by the SIBioc work group, who suggest establishing a multidisciplinary committee, assigned to: (i) stabilize the pertinence of POCT in the used setting (e.g., home or not); (ii) define the procedures for the telematic connectivity between the POCT and core laboratory; (iii) execute the digital localization of the “in-use” POCT; (iv) ensure compliance with the specific laws and rules [11]. Other important efforts are being made in regard to the improvement of the connectivity. Connectivity is defined as a process that enables the POCT devices to connect with the lab or hospital’s information. Such a system automatically validates and transfers POCT-derived results to the electronic medical record, aiding the monitoring and management of the data, POCT devices, and operators. Connectivity is critical to the successful implementation of a POCT service. In particular, it will make the LOCs use in clinical setting more efficient and cost effective [95].

Hence, the work involved in the implementation of a new POCT pathway can appear monumental and involves a transformation of diagnostic services and care provision [21,22,23].

Thousands of POCT devices have been developed in academic labs, but only a minority are able to analyze untreated samples and involve processes that make them suitable for home use. A small percentage of these devices have been commercialized and only a few of these have been successfully evaluated and integrated into clinical practice [24]. With the improvement of connectivity and the opportune distribution of the POCT-associated responsibility, it will be possible to strongly increase the use of POCT in hospital units, as well as in home applications.

## 6. Conclusions

CKD is an increasing health problem with high associated healthcare costs. CVD is the major cause of death in CKD patient. The possibility to monitor CKD patients by means of non-invasive and low-cost technologies will improve CKD management, patients’ quality of life, as well as optimizing expenses for CKD. In recent years, an increasingly patient-centered approach, as well as the recent coronavirus pandemic, has underlined the need to move from traditional lab-centralized diagnostics to LOC settings. Currently, many applications of LOCs in clinical setting exist. However, few devices are applied for CKD management. This is due to a dual reason: (i) some biomarkers useful for CKD management are quite difficult to quantify by POCT due to possible interferents; (ii) there is a need to amplify the biochemical markers panel to improve CKD patients’ quality of life. In this regard, monitoring the uremic toxins and inflammatory markers that are strictly associated to CVD complications in CKD will allow for better and earlier supervision of CKD progression. On the other hand, very few available POCT for CKD are for home-use. Indeed, it is important to underline that, irrespective of the ease of use of POCT devices, some of them can be used only by specialized persons. Alternatively, it is also important to be well-trained before the home-use of POCTs. Indeed, despite POCTs being designed to be relatively simple and low risk to use, they are not error-proof. Individuals using POCT devices, even healthcare personnel, must carefully follow the test directions and be familiar with the LOC system. To overcome these issues, many efforts are being made regarding the validation of some biomolecules, such as lanthionine, AGEs, and other uremic toxins, as markers of CKD progression; on the other hand, various research groups are working hard to design and develop adequate LOC systems that are able to measure uremic solutes.

Taking into account both the technological/industrial and clinical features, the most important aspects of the implementation of LOCs system in the clinical setting are: (i) a balance between the choice of materials, the suitable techniques of production, the costs, and the yield; (ii) method performance and validation; (iii) quality assurance; (iv) staff/user training; (v) the need to draw-up univocal guidelines for LOC use and management; (vi)the implementation of a panel of biomarkers to detect. The integration and interaction of biochemistry and medicine with microfluidic technology, on-chip electronics, and analytical chemistry are needed for addressing these issues.

## Figures and Tables

**Table 1:** “ASSURED” Guidelines for POCTs Development.

	Features	Specification
A	Affordable	Few than 10 dollars for Test—few than 500 dollars for machines
S	Sensitive	Minimal False Negative
S	Specific	Minimal False Positive
U	User-Friendly	Little training, easy to use
R	Rapid and Robust	Few than 30 min for result, minimal consumables, shelf life greater than one year at room temperature, high-throughput
E	Equipment-Free	Compact, on-site data analysis, battery powered
D	Delivered	Portable, Handheld

**Table 2:** Main Features of the detection methods used in LOC devices application.

Method	Detection Measurement	Advantages	Disadvantages
Electrochemical	Variations in electrical parameters such as conductance, resistance or capacitance	Rapid detection, low costs of fabrication	Short shelf-life, matrix interferences, need to control ionic concentration before measurement
Mechanical	Variations in resonant frequency or surface stress of the mechanical sensor	Label free detection, monolithic sensing integration	Very slow detection time, complex fabrication
Optical	Variations in absorbance, turbidity, fluorescence, refractive index	Rapid detection, no sample preparation	Optical instrumentation is generally expensive, complex set-up

**Table 3:** CKD staging, according to GFR and Albuminuria.

		A1	A2	A3
		<30 mg/gCr	30–300 mg/gCr	>300 mg/gCr
**G1**	>90 mL/min/1.73 m^2^	low	moderate	high
**G2**	60–89 mL/min/1.73 m^2^	low	moderate	high
**G3a**	45–59 mL/min/1.73 m^2^	moderate	high	very high
**G3b**	30–44 mL/min/1.73 m^2^	high	very high	very high
**G4**	15–29 mL/min/1.73 m^2^	very high	very high	very high
**G5**	<15 mL/min/1.73 m^2^	very high	very high	very high

G1, 2, 3a, 3b, 4 and 5 represent the GFR range used for CKD classification. A1, 2, 3 are the albuminuria range. Albumin concentration is expressed as mg of albumin per g of creatinine. “Low”, “moderate”, “high” and “very high” are referred to kidney function reduction.

**Table 4:** Examples of POCT devices, commercially available, useful for monitoring biochemical parameters in CKD patients.

	Device	Test	Use at Home	Ref.
1	Nova biomedical StatSensor and StatSensor Express cretinine	Creatinine and calculation of eGFR	No	[78]
2	Hemocue Albumin 201	Urinary Albumin	No	[79]
3	Roche Diagnostics CoaguChek XS	Prothrombin time and INR	Yes	[80]
4	Siemens Healthcare Diagnostics Xprecia Stride	Prothrombin time and INR	No	[81]
5	Entia Luma	Hemoglobin	Yes	[23]
6	EKF Diagnostics HemoControl	Hemoglobin and estimated Hematocrit	No	[82]
7	Abbott Laboratories FreeStyle Libre	Glucose-oxidase enzyme-based sensor	Yes	[83]

## Data Availability

Not applicable.
